# Decomposing socioeconomic inequalities in self assessed health in Turkey

**DOI:** 10.1186/1475-9276-11-73

**Published:** 2012-12-05

**Authors:** Kaan Sözmen, Hakan Baydur, Hatice Simsek, Belgin Ünal

**Affiliations:** 1 Narlidere Community Health Center, Ministry of Health of Turkey, Ilıca Mah.Güvendik Sk. No:5, Izmir, Narlıdere, 35320, Turkey; 2 Vocational School of Health Services, Celal Bayar University, Manisa, Turkey; 3 Department of Public Health, Dokuz Eylul University Faculty of Medicine, Izmir, Turkey

**Keywords:** Self assessed health, Socioeconomic inequality, Decomposition method, Turkey

## Abstract

**Introduction:**

This study aimed to measure socioeconomic inequalities in Self Assessed Health (SAH) and evaluate the determinants of such inequalities in terms of their contributions amongst the Turkish population.

**Methods:**

We used data from the Turkish part of World Health Survey 2003 with 10,287 respondents over 18 years old. Concentration index (CI) of SAH was calculated as a measure of socioeconomic inequalities in health, and contributions of each determinant to inequality were evaluated using a decomposition method.

**Results:**

In total 952 participants (9.3%) rated their health status as either bad or very bad. The CI for SAH was −0.15, suggesting that suboptimal SAH was reported more by those categorised as poor. The multiple logistic regression results indicated that having secondary, primary or less than primary school education, not being married and being in the lowest wealth quintile, significantly increased the risk of having poor SAH. The largest contributions to inequality were attributed to education level (70.7%), household economic status (9.7%) and geographical area lived in (8.4%).

**Conclusion:**

The findings indicate that socioeconomic inequalities measured by SAH are apparent amongst the Turkish population. Education and household wealth were the greatest contributing factors to SAH inequality. These inequalities need to be explicitly addressed and vulnerable subgroups should be targeted to reduce the socioeconomic disparities.

## Introduction

Self-assessed health (SAH) is widely used in epidemiological studies and it is well known as an important predictor of morbidity, mortality and health services utilisation [1,2]. It is also a marker of wellbeing and quality of life, which integrates individuals’ health conceptions and comparisons with health-related references. SAH also seems to be associated with socio-demographic, socio-economic, behavioural, psychosocial and chronic health conditions [3-6]. This has also been found when evaluating health inequalities in the population [7].

Moreover, Turkey is one of many developing countries experiencing rapid epidemiological transition, which involves dealing with both communicable and non-communicable diseases at the same time. This can also be referred to as the double burden of diseases [8]. Rapid changes in socioeconomic determinants of health continue to create disparities in health. Equity can be defined as “the absence of potentially remediable, systematic differences in one or more aspects of health across socially, economically, demographically, or geographically defined population groups or subgroups” (International Society for Inequity in Health – ISEqH) [9].

One of the main goals of national health systems is to reduce health inequalities so that disadvantaged groups may offer great potential for improving the health status of the whole population [10]. Determining the magnitude of the problem, the causes of health inequalities and the distribution of these determinants across population groups could help policy makers to target vulnerable groups and reduce such inequalities.

In Turkey, indicators show that inequalities exist in the following areas: health status, provision of health services, education, and income level across regions, yet few studies focus on these inequalities [11]. Research into the determinants and distribution of SAH is also very limited, and the majority of the data available is from only one region of the country [12,13]. The current study aimed to measure the socioeconomic inequalities in SAH, and also attempted to evaluate the determinants of such inequalities in terms of their contribution to SAH. This was the first study of its type to be carried out in Turkey.

## Methods

### Source of data and study design

The data for this study came from the Turkish part of the 2003 World Health Survey (WHS), which was conducted in 69 countries, across all continents [14].

The target population was all adults aged 18 years or over who were dwelling in private households in Turkey. The survey was administered to a random sample of Turkish individuals. The sampling method was designed and applied by the Turkish Statistical Institute (TurkStat).

The sample design was based on a stratified Probability Proportional to Size (PPS), with a two-stage collection of equal-sized clusters, which amounted to a total of 12,000 households. Sampling was conducted in 5 regions, and in rural versus urban locations within each region, in Turkey. The TurkStat sampling branch (formerly known as the State Institute of Statistics) selected 480 sample blocks consisting of 25 households. They used a systematic sampling technique in a digital environment, and households were selected with equiprobability employing an inverse sample design. Respondents to the personal questionnaire were typically aged eighteen years or more. They were selected from the list of households, in accordance with the Kish Table used for that specific household [15].

The World Health Surveys were approved by WHO’s ethical review process. Interviewers obtained informed consent for the survey, in writing, from the respondents. In this study we used dataset with no identifiable information on the survey participant from WHS which is publicly available upon request from WHO [16].

### Dependent variable

Self-assessed health (SAH) was the outcome variable. It was evaluated by asking about the current health state of respondents, with the possible answers being “very good” (1), “good” (2), “moderate” (3), “bad” (4) or “very bad” (5). Responses were classified as a dichotomous measure: “very good”, “good” or “moderate” were coded as optimal SAH, and “bad” or “very bad” as poor health status.

### Independent variables

The independent variables used in this survey were age, gender, level of education, marital status, location of residence (urban/rural), specific province of residence, having a chronic disease, and wealth.

Participants’ ages at the time of interview were categorized into six age groups (18–25 years; 26–35 years; 36–45 years; 46–55 years; 56–65 years, and ≥66 years). Respondents were asked to report their highest level of education completed, for which responses were grouped as “no formal schooling or less than primary school”, “primary school completed”, “secondary school completed”, “high school (or equivalent) completed and college/pre-university or university completed”. Marital status was classified as single, married, widowed, divorced or separated. Province of residence was divided into 5 groups (Western, Black Sea, Mediterranean, Middle and Eastern), as well as into urban and rural areas. If an individual had at least one long-term condition such as arthritis, angina, asthma or depression they were categorised as having a chronic disease.

Summing expenditures from the previous four weeks on food, education, housing, health care, insurance premiums and ‘other’ constructed the wealth variable. In order to correct for household size, this number was divided by the square root of the household size. This proxy for wealth was used as an indirect measure of socioeconomic status [17].

### Statistical analysis

In order to avoid biasing effects, we took into account the stratification and the unequal sampling weights from the WHS and we used the survey (svy) estimation techniques provided by STATA 11.0.

Concentration index (CI) is used as a measure of socioeconomic inequality and is derived from the concentration curve (CC). The CC plots the cumulative percentage of the health variable on the y-axis, against the cumulative percentage of the population, ranked by socioeconomic status, starting with the poorest and ending with the richest on the x-axis. If everyone, irrespective of socioeconomic status, has exactly the same value on the health variable, the CC will be a 45-degree line, which is also called the “line of equality”. The CI is computed as twice the area between the CC and the line of equality. The CI takes values between interval −1 and 1 and is related to the extent of prevailing socioeconomic inequality. If the health variable (i.e. poor SAH) is more concentrated amongst the poor, the concentration curve would lie above the line of equality and CI will have negative values [18]. In other words, a negative (positive) value of CI means that poor SAH is higher among the poor (rich). The interpretation of the magnitude of a CI value is not straightforward. Multiplying the CI value by 75 gives an estimation of the percentage of the variable of interest (SAH) to be redistributed from the richer half to the poorer half, in order to reach a distribution of perfect equity and to obtain a CI value of zero [19].

The Concentration index can be written as follows:

(1) $$CI=\frac{2}{\mu }COV_{w}\left(yi,Ri\right)$$

Here, y_i_ refers to the level of the health variable of ith individual, Ri is the fractional rank of the ith ranked individual in the socioeconomic distribution in terms of income, whilst the μ is the weighted mean of y, and cov_w_ is the weighted covariance.

### Decomposing socioeconomic inequality

The exact contribution of each of the explanatory factors of the observed socioeconomic inequality can be estimated by decomposing the CI into the pre-specified explanatory factors, together with an unexplained component (ε_i_). This can be done by running a linear regression on all individuals in a sample, explaining poor SAH, y_i_, with a set of explanatory variables, k [17].

(2) $$y_{i}=\alpha +\sum_{\mathrm{ki}}\beta_{\mathrm{ki}}X_{\mathrm{ki}}+\varepsilon_{i}$$

Here βk represents the coefficient of the explanatory variables and εi is the error term. Given the relationship between y_i_ and x_ki_ in Equation 2, the concentration index for yCI can be rewritten by combining equations 1 and 2 as in equation 3.

(3) $$yCI=\sum_{k}\left(\frac{\beta_{k}x_{k}}{\mu }\right)CI_{k}+\frac{GCI_{\varepsilon }}{\mu }$$

Equation 3 shows that the overall inequality in health outcome has a deterministic or "explained" component and an "unexplained" component, which cannot be explained by systematic variation. In the former, the βk component is the coefficient from a regression of health outcome on a determinant k, ${\overset{⌣}{x}}_{k}$ is the mean of this determinant k, μ is the mean of the health outcome, and CIk is the concentration index for the determinant k. In the latter component, GCIε is the generalized CI for the error term. The contribution of each determinant to inequality can be quantified through multiplying the health variable elasticity with respect to that determinant and its concentration index $\left(\frac{\beta_{k}x_{k}}{\mu }\right)\;CI_{k}$.

In summary, the contribution is determined by both the effect of the explanatory variable on the outcome, as well as its distribution by economic status, as represented by the CIs. Even if the impact of the determinant on the outcome is large, if it is equally distributed across income groups, it will not be the main driving factor, which explains socioeconomic inequalities in health.

The percentage contribution of each determinant can be calculated simply, through dividing its absolute contribution by the CI of the health variable $\left(\frac{\beta_{k}x_{k}}{\mu }\right)CI_{k}/CI$.

(4) $$h_{i}=\alpha^{m}+\sum_{k}\beta_{k}^{m}x_{\mathrm{ki}}+u_{i}$$

The decomposition method was initially introduced for using with linear prediction models [18]. However, in our study the multivariate analysis of binary dependent health variable (i.e. poor SAH) requires nonlinear estimation methods, such as a logit model, which has been used in other studies [7]. In a logit model, using marginal effects (dh/dx) allows for dealing with discrete changes from 0 to 1, which give the change in predicted probability associated with unit change in an explanatory variable. This then restores the mechanism of the decomposition framework in equations 2 through 4 [20]. In the present study, all variables were included in the logit model in order to calculate the adjusted odds ratios. Explanatory variables were also included as dummy variables in the decomposition analysis model. The marginal effects obtained from the logistic regression analysis indicate an association between the determinants and the dependent variable (poor SAH). Marginal effects with positive signs are indicative of positive associations with the probability of reporting a health outcome, whilst those with negative signs indicate negative associations. Also, the larger the absolute value of a marginal effect the more substantial the association. The data were analysed with STATA/SE 11.0 software (StataCorp, College Station, TX, USA). The level of statistical significance was set to 0.05.

## Results

**Figure 1:**
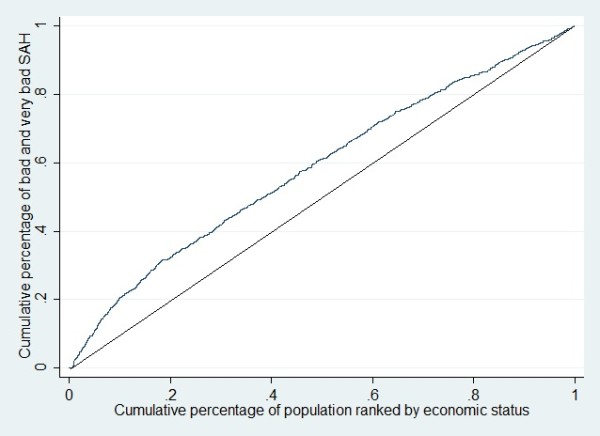
Concentration curve for poor self assessed health.

The survey was administered to 11,481 (95.7%) of the target sample. After excluding cases with missing values, analyses were conducted for 10,287(89.6%) individuals in total. Overall, 952 participants (9.3%) rated their health status as bad or very bad. The CI for poor SAH was −0.15 (SE 0.03). Figure 1 illustrates the CC of SAH, indicating that poor health is reported more by less wealthy individuals. The required redistribution of an optimal SAH from the richer to the poorer half of the population in order to obtain zero inequality was 11.5%.

Table 1 presents summary statistics, crude and adjusted odds ratios between self-assessed health (SAH) and determining factors. The numbers outside the brackets indicate sample counts and the numbers inside the brackets represent “population-weighted” percentages. For both crude and adjusted analyses, women had significantly worse SAH in comparison to men (Table 1). Nonetheless, age, level of education, marital status, and chronic disease were significantly associated with poor SAH in the unadjusted analyses. In the multiple logistic regression, the chance of poor SAH among the widowed, divorced-separated respondents with secondary, primary or less than primary school education were significantly higher. Amongst those in the richest wealth quintile, 6.5% rated their health status as bad or very bad, whilst this figure was 13.3% among those in the poorest quintile. The probability of having poor SAH also got higher with increasing age over 36–45 years old after controlling for other explanatory factors. Individuals residing in the Eastern and Middle part of the country had higher rates of poor SAH, but no significant difference was found between urban–rural residence.

**Table 1:** Summary statistics, crude and adjusted odds ratios between self-assessed health (SAH) and determining factors

**Variables**	% Poor SAH*	**Crude OR (95% CI)**	**Adjusted OR (95%CI)**
Gender			
Male	5.4(261/4428)	1	1
Female	10.7(691/5859)	2.08(1.66–2.59)	1.44(1.12–1.86)
Age, years			
18-25	3.1(62/1682)	1	1
26-35	5.6(155/2547)	1.86(1.24–2.78)	1.51(0.95–2.42)
36-45	8.4(193/2317)	2.87(1.87–4.40)	1.91(1.18–3.08)
46-55	8.7(160/1564)	2.96(1.98–4.43)	1.65(1.01–2.72)
56-65	14.3(157/1076)	5.22(3.29–8.28)	2.38(1.39–4.09)
66+	19.2(225/1076)	7.40(5.11–10.74)	2.80 (1.67–4.69)
Marital Status			
Married	8.2(686/7874)	1	1
Single	3.1(46/1342)	0.36(0.23–0.54)	0.86 (0.52–1.42)
Divorced-Seperated	22.8(193/921)	3.32(2.61–4.22)	1.73 (1.03–2.92)
Widowed	12.9(27/150)	1.66(1.05–2.64)	1.31(0.96–1.78)
Educational Status			
High School-University	1.5(16/833)	1	1
Secondary	3.1(83/2485)	2.07(1.05–4.09)	2.19(1.10–4.32)
Primary	7.6(372/4735)	5.31 (2.80–10.45)	4.28(2.22–8.24)
Less than primary	20.1(481/2234)	16.36 (8.69–30.79)	8.73(4.52–16.87)
Setting			
Urban	8.2(445/5202)	1	1
Rural	8.7(507/5085)	1.07(0.86–1.34)	1.03(0.84–1.27)
Chronic Disease			
No	6.4(588/8253)	1	1
Yes	17.2(364/2034)	3.06(2.54–3.69)	2.12(1.70–2.65)
Wealth Quintiles			
5th(richest)	6.5(106/1583)	1	1
4th	7.0(119/1772)	1.04(0.71–1.51)	0.90(0.83–1.75)
3th	7.4(160/1976)	1.16(0.84–1.60)	0.95(0.55–1.12)
2nd	7.7(203/2287)	1.35(0.99–1.83)	0.79(0.65–1.38)
1st(poorest)	13.3(364/2669)	2.21(1.62–3.01)	1.21(0.60–1.38)
Geographic characteristics			
Mediterranean	6.1(84/1321)	1	1
West	7.1(233/3169)	1.18(0.81–1.73)	1.29(0.89–1.88)
Black Sea	8.7(111/1266)	1.46(0.99–2.16)	1.41(0.97–2.07)
Middle	10.6(174/1649)	1.83(1.24–2.71)	1.89(1.29–2.80)
East	11.6(350/2882)	2.02(1.42–2.88)	1.91(1.34–2.72)
Self Assessed Health,%(n)	8.4(952/10287)		

Table 2 presents the results of the decomposition of the SAH CI, through combining the estimated logit coefficients with information on the means of dichotomized variables, CIs and marginal effects of the explanatory variables. The CIs demonstrate that persons aged 65 or over were strongly concentrated among the poor (CI = −0.143). Those who were widowed (CI = −0.142) as well as individuals with less than primary education level (CI = −0.221) were also more prevalent among the poor. Individuals of working age (46–55) (CI =0.070) and those with at least high school education level (CI = 0.424) tended to be better off. People residing in the western part of the country also seemed to be relatively well off (CI = 0.095), but the opposite was true for those living in the east (CI = −0.186). Chronic diseases were also slightly more concentrated among the poor (CI = −0.005).

**Table 2:** Decomposition results for socioeconomic inequality

**Variables**	**Weighted mean**	**Marginal effect**	**Concentration index(CI)**	**Contribution (CI_SAH_ = −0.145)**	**Percentage contribution**	**Per category (%)**
**Gender**						2.9
Female	0.059	0.119*	−0.037	−0.004	2.9	
**Age, Years**						
26-35	0.227	0.025	−0.029	−0.002	1.2	4.9
36-45	0.209	0.042*	0.064	0.007	−4.0	
46-55	0.171	0.032	0.070	0.005	−2.7	
56-65	0.104	0.064*	−0.081	−0.006	3.9	
66+	0.092	0.082*	−0.143	−0.013	7.7	
**Marital Status**						
Single	0.169	−0.007	0.069	−0.001	0.6	1.7
Divorced-Separated	0.010	0.037	0.022	0.001	−0.1	
Widowed	0.071	0.016	−0.142	−0.002	1.2	
**Educational Status**						
Secondary	0.254	0.051	0.181	0.028	−16.8	70.7
Primary	0.467	0.087*	−0.029	−0.032	19.4	
Less than primary	0.195	0.220*	−0.221	−0.113	68.1	
**Setting**						0.1
Rural	0.385	0.002	−0.017	0.000	0.1	
**Chronic Disease**						0.3
Yes	0.188	0.051	−0.005	0.000	0.3	
**Wealth Quintiles**						
4th	0.195	−0.005	0.418	−0.004	2.9	9.7
3th	0.204	−0.003	0.060	0.000	0.2	
2nd	0.203	−0.012	−0.357	0.010	−6.2	
1st(poorest)	0.201	0.011	−0.831	−0.021	12.8	
**Geographic residence**						
West	0.460	0.014	0.095	0.007	−4.4	8.4
Black Sea	0.108	0.021	−0.004	0.000	0.1	
Middle	0.176	0.042*	0.022	−0.007	4.7	
East	0.138	0.043*	−0.088	−0.013	8.0	
**Total sum contribution**				**−0.166**	100	100
Residual(Unexplained)				0.021		

*p < 0.05.

Reference groups were males, age group 18–25, married, high school-university, urban, no chronic disease, wealth quintile 5(richest); geographic residence:Mediterranean.

Moreover, Table 2 shows the marginal effects of each determinant on poor SAH. The relationship between low education level and poor SAH is evident. The interpretation of this result was after controlling for all other variables. Being in the less than primary education level increases individuals’ probability of having poor SAH by approximately 22.0 percent (marginal effect = 0.220, p < 0.05). Individuals with chronic disease had a 5 percent higher risk of having poor SAH. Likewise, those aged 66 and over had a increased risk of poor SAH (8.2 percent), whilst individuals aged between 55 and 65 showed a 6.2 percent greater risk of poor SAH, and 4.2 percent higher for those individuals residing in the eastern region.

The absolute contribution of each determinant was obtained by multiplying its marginal effect by its mean and CI, then dividing it by the mean of the health outcome. For example, the contribution of having a less than primary education can be computed as: Marginal effect (0.219902)*Mean (0.195276)*CI of x(−0.22143) divided by weighted mean of SAH (0.084) = −0.113. Percentage contribution was computed by dividing the contribution of each determinant by the total explained portion of the CI (−0.166), which is = −0.113/-0.166 = 68.1%. Therefore, having less than primary education contributes to 68% of the inequalities in SAH.

The main contributors to inequality in SAH were: level of education (70.7%), wealth (9.7%) and geographical area lived in (8.4%). Gender and marital status played a less important role in terms of inequalities.

## Discussion

The results of the current study revealed for the first time, evidence of the composition of socioeconomic inequality in Turkey, employing CI in SAH. Total CI for poor SAH was negative (−0.15), which implies that poor SAH was more common among the less wealthy. The results also show an increase in poor SAH with older age, and an inverse significant relationship was found with level of education. Individuals who were separated, divorced or widowed, residing in the eastern or middle part of the country and those with chronic diseases had significantly worse SAH values. We assessed inequality through decomposition analysis, which takes into account both unequal distribution of the determinant and effect of the determinant on poor SAH. Decomposition results revealed that whilst household economic status, education level and geographic area lived in contributed to around 89 percent of the total socioeconomic inequalities in having poor SAH. However, gender, marital status, living in a rural area and having a chronic disease did not have high impact on inequalities.

Furthermore, we found that a higher proportion of women reported poor health compared to men, a finding in line with research from other developing and developed countries [3,4,21]. According to the 2008 Turkish Health Survey, 7.0% of men and 13.2% of women declared their general health status as either bad or very bad [22]. A study from the Netherlands conducted amongst various ethnic groups found that Turkish women showed poorer health than men [23]. In the current study, gender contributed to 3% of the inequalities in SAH. We expected that the contribution of gender differences to inequalities would be higher. As per this finding, perhaps gender is not directly associated with SAH, but is related to other variables like education, wealth, marital status and health related issues, which play an intermediate role in SAH inequalities. For example, although there has been a significant improvement in overall literacy in Turkey since 1990, 19.4% of women were illiterate compared to the 6.1% of men, which might influence the social status of women. In addition, economic dependence, marital status, and family demands also contribute to gender differences in SAH [24]. A Turkish study showed that the health status of women is poorer, which can at least be partially associated with the reproductive period, where major health risks are faced by women, but not by men [25]. Also, factors such as obesity, depression, musculoskeletal system disorders and difficulty in performing personal care are also more prevalent among women in Turkey [22], which could partly explain the gender differences in SAH.

We also found that age was a significant determinant of poor SAH for both univariate and multivariate analyses. Being in the 66 and over age group contributed to 7.7% of inequalities in SAH. Evidence from other studies has shown similar findings regarding the effect of aging on SAH [4,22,26]. In our study, marginal effects were positive across all age groups, but the pro-rich distribution among the middle aged groups (36–45, 46–55) represented with positive CIs, decreased the magnitude of the overall contribution of age. This finding is similar to the results of an Iranian study [27].

Those who were divorced, separated or widowed had significantly higher odds of poor SAH than their respective reference group in an unadjusted logistic regression. This result is in accordance with previous research findings [27]. In the multiple logistic regression, only being divorced remained significant. Being divorced may have more health impacts than being widowed, especially amongst women. This may be because divorced women appear to have more financial problems, psychological distress and health damaging behaviours, which could have negative effects on SAH [28]. In Turkey, nearly 75% of the women are housewives and do not form part of the labour market through paid jobs. The majority of women do not have a formal employment history that make them eligible for retirement [29]. Therefore, in the case of divorce or death of the spouse, who is informally employed, women are forced into poverty and deprivation more because of the lack of both income and social security, which could affect their health status. Marital status contributed only slightly to inequalities in poor SAH, whilst being widowed contributed more to inequalities compared to other categories. This same result was also found in another study [27].

Moreover, individuals who reported at least one chronic condition had twice the adjusted odds of having poor SAH, which is in line with other research findings [7,26]. In contrast, chronic disease had a small contribution to inequalities in SAH, which is the result of a very small CI value (−0.005), indicating that chronic diseases were slightly more concentrated among the poor. One possible explanation for this could be the underreporting of long term conditions of less wealthy individuals, due to poor access to health care services, resulting in low awareness rates [30]. According to the 2003 Turkish National Health Accounts Survey, less wealthy individuals reported lower rates of chronic conditions and higher rates of poor SAH, compared to wealthy individuals [24].

In our study, low level of education was the main determinant of inequalities in SAH. Analysis of the disaggregated dataset from WHS from 69 countries showed that adults with lower levels of education were consistently more likely to report poor health than those with higher levels of education. This finding was not dependent on a country’s level of economic development or geographical region [31]. A study using WHS data from Brazil indicated that a low degree of education was associated with poor SAH in a multivariate analysis [32]. A study from Greece also found that individuals with less education were more likely to rate their health as worse [33]. In a recent analysis of WHS data using a relative index of inequality from 57 countries, it was reported that the education-related inequality in poor health was higher than the wealth-related inequality. This finding is concordant with the findings of the present study [34]. An inverse association between level of education and SAH has also been reported in previous studies [3,4,7,35]. Education is likely to be a determinant of socioeconomic indicators such as economic status, occupation and lifestyle. Individuals with a high level of education are more aware of their health conditions and have better access to health care services. Therefore, education has a significant impact on the observed inequalities in health amongst different socioeconomic subgroups of the population [36]. In our case, the combination of the negative effect of low education on SAH and pro-poor distribution (CI = −0.221) of low level of education made a substantial contribution to inequalities in SAH in Turkey.

In this study, a positive sign of marginal effect in the lowest income quintile indicated a positive association for reporting poor SAH in this income group. The chance of having poor SAH was 2.2 times higher in the poorest group, compared to the richest income quintile in the unadjusted analysis. A similar finding emerged in a previous study [27]. This finding is also concordant with the Demographics and Health Survey Turkey, which reported a 3.5 times greater infant death rate in the poorest quintile compared to the richest [37]. In our study, when other factors such as education were included in the model, the effect of income on poor SAH substantially reduced and lost its statistical significance. In Turkey, education is considered a proxy for income; usually individuals with higher education also have higher incomes. Education could be a better indicator than some of the social factors like income, linked to social position, which are important for health [38].

In terms of geographical location, residing in the Eastern part of Turkey was one of the main contributors to inequalities in SAH. The eastern region has the lowest income per capita as well as a negative socio-economic development index, comprising indicators of demographic structure, labour demand, quality of education and health services, in comparison to other regions [39]. This region also has the birth and infant death rates; in 2008 only 1.6% of infants in the west of Turkey died before age one, compared to 3.9% of infants in the east [37]. The proportion of women attending school ranged between 78.0-80.0% in the east, whilst this figure was around 94% in the west, in 2004 [40]. According to the National Health Accounts Survey, the majority of people (64%) living in the eastern region were on the Green Card scheme that covers people without any health insurance, whilst in the west only 35% of people were on this scheme in 2003 [24]. The percentage of people who took no action to overcome a health problem once it occurred was higher compared to other regions, due to a lack of money, long distances to health care facilities or a lack of quality services in the east [24]. Poor SAH was more common amongst people living in rural areas, but no significant relationship emerged from the logistic regression analysis. This could mean other risk factors like education and wealth were more important and diluted the effect of place of residence.

### Methodological considerations and limitations of the study

Even though SAH is a good, valid indicator of health, evidence shows that there are differences in reporting health between socioeconomic groups. It has also been found that the impact of health problems on SAH is stronger amongst better educated individuals, which could lead to the underestimating of health inequalities [41]. Whilst in developed countries the association of SAH and mortality was stronger in the higher socioeconomic and education categories, a study from a developing country indicated that individuals with a lower level of education were more likely to report poor health [42]. These findings suggest that assessment with SAH could lead to an under or overestimation of the magnitude of existing health inequalities between socioeconomic groups in different countries. In contrast, reports from developing countries show evidence of the effectiveness of SAH measures, and that it is unlikely to be misleading when used in inequality research, as some have claimed [43].

Furthermore, when conducting the analyses we removed individuals with any missing data on key variables, which may introduce bias, but there was no statistically significant difference in the distribution of key parameters such age, gender, education, SAH and income amongst individuals with and without (results available from the authors) complete information. We generated chronic disease variable by using only four long term conditions that the dataset was involving. As a result the long term conditions that were not assessed in the questionnaire might have some impact on the magnitude of inequalities and relationship with SAH, however we believe this have a limited effect on our results.

The design of the study was cross-sectional; hence, inferring causality in measured factors requires caution. The decomposition method is a deterministic approach and there could be other factors, which were not included in the model, that may contribute to the inequalities in SAH, such as cultural and health system related determinants. In particular, we were unable to evaluate access to health care and its contribution to inequalities. The data is self-reported, which could lead to information and recall bias. We used expenditures as a proxy for socioeconomic status, which implies that there is a possibility of reporting bias. In developing countries, formal employment is less common and many households have multiple and changing sources of income. Therefore, it is generally easier and more reliable to measure consumption than income in our settings [17]. Another limitation is the potential impact of choice of proxy measure for socioeconomic status on the study findings. Some research has also found inconsistencies in measured inequalities, when different socioeconomic measures were used on the same data[44]. However, two other studies found that the choice of welfare indicator made little difference to the measured degree of socioeconomic inequalities [45,46]. The present study used expenditure data to create a living standards variable in order to generate a concentration index. There is also a consensus among economists that expenditure is the preferred measure of socioeconomic status, rather than income, since it has more of a theoretical basis[47].

### Policy implications

During the last decade, Turkey has shown important social and economic improvements. Although infant mortality rates decreased from 38 to 19 per thousand and health insurance coverage increased from 66% to 77% between 2000–2008, inequalities still remain across regions and income quintiles [37,48]. For example, according to the 2010 OECD data, the top 20% income quintile of the adult population in Turkey rated their health as ‘good’ or ‘very good’, compared to 59% for the bottom 20% [49]. These disparities between socioeconomic groups are unfair and avoidable. The main principles used to tackle inequalities are: focusing on the most disadvantaged groups, narrowing health gaps and reducing social gradients [50]. Even though interventions seem hard to implement, there are good examples from developing countries that target social determinants of health. In Brazil, a conditional income programme called Bolsa Familia has addressed the interconnected conditions that lead to poor and inequitable health [51]. The programme deals with key aspects such as child development through education and health services, employment through vocational training, and giving support to family agriculture to secure income and reduce poverty. Families must ensure that children attend school regularly and that their vaccination status is monitored to receive support. The programme is considered to be one of the factors that led to a more equitable income distribution across the country [52]. We believe that merging the country-specific evidence with good practices from the developing world could help policy makers to create equitable health development interventions. Hence, it will be beneficial to assess the effects and contributions of socioeconomic determinants of self assessed health in various countries by future surveys using similar methodology which will allow evaluating variations across countries.

## Conclusion

The findings of the study reported here provide evidence for the existence of socioeconomic inequalities in self-assessed health in Turkey. Our results indicate that socioeconomic inequality in SAH is mainly determined by factors beyond the scope of health system such as: education, household wealth and geographical area lived in. In this respect, individuals with a lower education level, low income and living in eastern regions should be seen as the priority target populations. In order to tackle health inequalities and improve overall health in Turkey, greater emphasis should be placed upon providing equal access and opportunities for education and employment amongst these vulnerable groups. We believe that our findings will be useful for policymakers, who are faced with the task of reducing social inequalities.

## Competing interests

The authors declare that they have no competing interests.

## Authors' contributions

KS conceived the study, data analysis, statistical analysis and drafted the manuscript. HB made substantial contribution to analysis and interpretation of data. HS and BU contributed to the interpretation of results and final writing of the paper. All authors read and approved the final manuscript.
